# A high mean arterial pressure target is associated with improved microcirculation in septic shock patients with previous hypertension: a prospective open label study

**DOI:** 10.1186/s13054-015-0866-0

**Published:** 2015-03-30

**Authors:** Jing-Yuan Xu, Si-Qing Ma, Chun Pan, Hong-Li He, Shi-Xia Cai, Shu-Ling Hu, Ai-Ran Liu, Ling Liu, Ying-Zi Huang, Feng-Mei Guo, Yi Yang, Hai-Bo Qiu

**Affiliations:** Department of Critical Care Medicine, Nanjing Zhongda Hospital, School of Medicine, Southeast University, Nanjing, 210009 P.R. China; Department of Critical Care Medicine, Qinghai Provincial People’s Hospital, Xining, 810000 P.R. China

## Abstract

**Introduction:**

The effect of mean arterial pressure titration to a higher level on microcirculation in septic shock patients with previous hypertension remains unknown. Our goal is to assess the effect of mean arterial pressure titration to a higher level on microcirculation in hypertensive septic shock patients.

**Methods:**

This is a single-center, open-label study. Hypertensive patients with septic shock for less than 24 hours after adequate fluid resuscitation and requiring norepinephrine to maintain a mean arterial pressure of 65 mmHg were enrolled. Mean arterial pressure was then titrated by norepinephrine from 65 mmHg to the normal level of the patient. In addition to hemodynamic variables, sublingual microcirculation was evaluated by sidestream dark field imaging.

**Results:**

Nineteen patients were enrolled in the study. Increasing mean arterial pressure from 65 mmHg to normal levels was associated with increased central venous pressure (from 11 ± 4 to 13 ± 4 mmHg, *P* = 0.002), cardiac output (from 5.4 ± 1.4 to 6.4 ± 2.1 l/minute, *P* = 0.001), and central venous oxygen saturation (from 81 ± 7 to 83 ± 7%, *P* = 0.001). There were significant increases in small perfused vessel density (from 10.96 ± 2.98 to 11.99 ± 2.55 vessels/mm^2^, *P* = 0.009), proportion of small perfused vessels (from 85 ± 18 to 92 ± 14%, *P* = 0.002), and small microvascular flow index (from 2.45 ± 0.61 to 2.80 ± 0.68, *P* = 0.009) when compared with a mean arterial pressure of 65 mmHg.

**Conclusions:**

Increasing mean arterial pressure from 65 mmHg to normal levels is associated with improved microcirculation in hypertensive septic shock patients.

**Trial registration:**

Clinicaltrials.gov: NCT01443494; registered 28 September 2011.

**Electronic supplementary material:**

The online version of this article (doi:10.1186/s13054-015-0866-0) contains supplementary material, which is available to authorized users.

## Introduction

Septic shock is a major cause of death in critically ill patients and is characterized by hypotension and hypoperfusion [1]. Although mortality has been reported to be decreased due to the progress in therapy for septic shock [2,3], microcirculation abnormalities can still be present and contribute to hypoperfusion [4]. Microvascular dysfunction is associated with organ dysfunction and mortality [5,6]; moreover, early improvement in the microcirculation during resuscitation is associated with reduced organ failure [7]. Therefore, therapeutic strategies aimed at improving the microcirculation are warranted to mitigate hypoperfusion and organ failure.

Maintaining sufficient perfusion pressure might be an important way to improve the microcirculatory perfusion [8]. As one of the key determinants of perfusion pressure, adequate mean arterial pressure (MAP) might reduce hypoperfusion and organ failure. Correa and colleagues [9] have evaluated the effects of different MAP targets in septic pigs, and show that targeting a MAP between 50 and 60 mmHg in septic shock is associated with increased incidence of acute kidney injury when compared with a target MAP of between 75 and 85 mmHg. Moreover, Badin and colleagues [10] have reported that acute kidney injury is decreased with a MAP of at least 72 mmHg. However, which MAP levels should be targeted to improve microcirculation remains controversial [11-13].

The optimal MAP target in septic shock patients, especially in patients with previous hypertension, is still unknown [14]. The guidelines [15] for treating septic shock have recommended that MAP should be maintained at a level of at least 65 mmHg, and emphasized a higher blood pressure for patients with previous hypertension. A recent published randomized controlled study [16] has shown no difference in mortality in septic shock patients undergoing resuscitation with a high MAP target or a low MAP target. However, among chronic hypertensive patients, those in the high-target group require less renal replacement therapy, indicating that septic shock patients with chronic hypertension might need higher MAP to maintain microcirculation.

Our goal was to assess the effect on the microcirculation of MAP titration to normal levels in septic shock patients with previous hypertension. We hypothesized that the increase in MAP from 65 mmHg to the normal levels of patients is associated with improved sublingual microcirculation.

## Materials and methods

### Setting

This was a single-center, prospective, open-label study conducted in the intensive care unit of a tertiary care teaching hospital. The study protocol was approved by the Ethics Committee (Approval Number: 2011ZDllKY03.0) of Zhongda Hospital, School of Medicine, Southeast University, and informed consent was given by each patient or their next of kin.

Trial registration: Clinicaltrials.gov NCT01443494; registered 28 November 2011.

### Study population

Patients admitted to the intensive care unit who met the following inclusion criteria were enrolled in the study: 1) patients with septic shock for less than 24 hours with a history of previous hypertension (septic shock was defined by the 2001 SCCM/ESICM/ACCP/ATS/SIS International Sepsis Definitions Conference [17]); 2) fluid resuscitation was performed according to the guidelines for treating septic shock to maintain the central venous pressure (CVP) at more than 8 mmHg and central venous oxygen saturation at more than 70%; 3) patients required norepinephrine (NE) to maintain a MAP of 65 mmHg. All patients were treated with mechanical ventilation, and received infusions of morphine and propofol. All patients had an arterial catheter and a central venous catheter in place.

Exclusion criteria were: pregnancy; age <18 years; inability to acquire the usual level of MAP; refusal of consent by the patient or relative; participation in other trials during the last 3 months; and hypertensive patients without antihypertensive therapy.

### Interventions

As chronic hypertensive patients are supposed to have undergone more blood pressure measurements in daily life than normotensive patients, the average MAP acquired from a patient’s physical examination records over the last 2 years was taken to be the target MAP. If the medical records were incomplete, a detailed enquiry about the target MAP to their next of kin was performed. In addition, age, gender, duration of shock prior to inclusion, Acute Physiology And Chronic Health Evaluation II score, intensive care unit length of stay, in-hospital mortality, source of infection and comorbidities were also recorded.

### Study design

After stabilization for 30 minutes, basal measurements including hemodynamic and microcirculatory measurements were taken 20 minutes apart, and the NE doses were increased to titrate MAP to the target level. Patients were allowed to stabilize for 30 minutes before new measurements were taken. Thus, there was 50 minutes between the baseline and the second measurement. During the process, no other changes in treatment were allowed. The decision to stop the research was made by the treating intensivist according to predetermined safety limits for each patient. The detailed safety limits are reported in Additional file 1.

### Hemodynamic measurements

Serial measurements of NE dose, MAP, heart rate and CVP were performed. Noninvasive bioreactance cardiac output and stroke volume monitoring were obtained using the NICOM system (Cheetah Medical, Portland, OR, USA). Arterial and central venous gases were obtained to determine arterial pH, hemoglobin, lactate, arterial partial pressure of oxygen, arterial partial pressure of carbon dioxide, arterial hemoglobin saturation, arterial oxygen partial pressure to fractional inspired oxygen ratio and central venous hemoglobin saturation.

### Microcirculatory measurements and analysis

Measurements of the sublingual microcirculation were obtained using sidestream dark field (SDF; Microscan, Microvision Medical, Amsterdam, The Netherlands). The SDF probe was placed under the sublingual area without pressure after removal of secretions. Video sequences of 20 seconds each were recorded from three different sublingual sites. These images were stored and later renumbered by an identifier blinded to the clinical course. Videos were converted to the AVI (audio video interleaved) file format with video processing software, and analysis for each image was performed by two different investigators. Finally, all the variables were averaged to yield a single value for statistical analysis. The microcirculatory flow index (MFI), based on determination of the predominant type of flow in four quadrants for small vessels and total vessels, was determined with a semi-quantitative methodology. Flow was characterized as no flow = 0, intermittent = 1, sluggish = 2, and continuous = 3, to reflect blood velocity, identical to that described in consensus conference recommendations [18]. MFI was calculated for all quadrants of the image and averaged for each sublingual site. Detailed methods are reported in Additional file 2.

### Statistical analysis

Statistical analysis of the data was performed using SPSS 16.0 (IBM, Somers, NY, USA). Histograms and normal-quantile plots were examined and Kolmogorov-Smirnov test was performed to verify the normality of the distributions of the data. Data are presented as the mean ± SD if normally distributed or median (interquartile range) if not normally distributed. Categorical variables are presented as number and percentage. For continuous data, paired *t* test or Wilcoxon signed-rank test were used. Differences were considered significant at *P* < 0.05.

## Results

### Clinical characteristics of study subjects

The main characteristics of the 19 patients are presented in Table 1. Detailed enquiry about the target MAP to their next of kin was performed for all of the patients. The averaged value of MAP was 93 mmHg in our study, which was maintained in the range 80 to 110 mmHg under the control of medications. The mean duration of shock prior to study inclusion was 16 hours. In-hospital mortality was 53%. The most common source of sepsis was pneumonia.

**Table 1:** **Baseline patient characteristics**

**Patient characteristic**	**(n = 19)**
Age (years)	74 ± 11
Gender (%)	13 males (68)
Duration of shock prior to inclusion (hours)	16 ± 6
APACHE II score	24 ± 10
Intensive care unit length of stay (days)	12 ± 8
In-hospital mortality (n (%))	10 (53)
Source of infection (n (%))	
Respiratory infection	11 (58)
Abdominal infection	6 (32)
Bacteremia	1 (5)
Urinary tract infection	1 (5)
Comorbidities (n (%))	
Chronic hypertension	19 (100)
Diabetes	2 (10)
Chronic renal failure	2 (10)
Usual mean arterial pressure acquired from previous medical records (mmHg)	93 ± 7
Fluid infusion during the study (ml)	185 ± 55

APACHE, Acute Physiology and Chronic Health Evaluation. Data presented as mean ± SD or n (%).

### Effect on global hemodynamics

**Figure 1:**
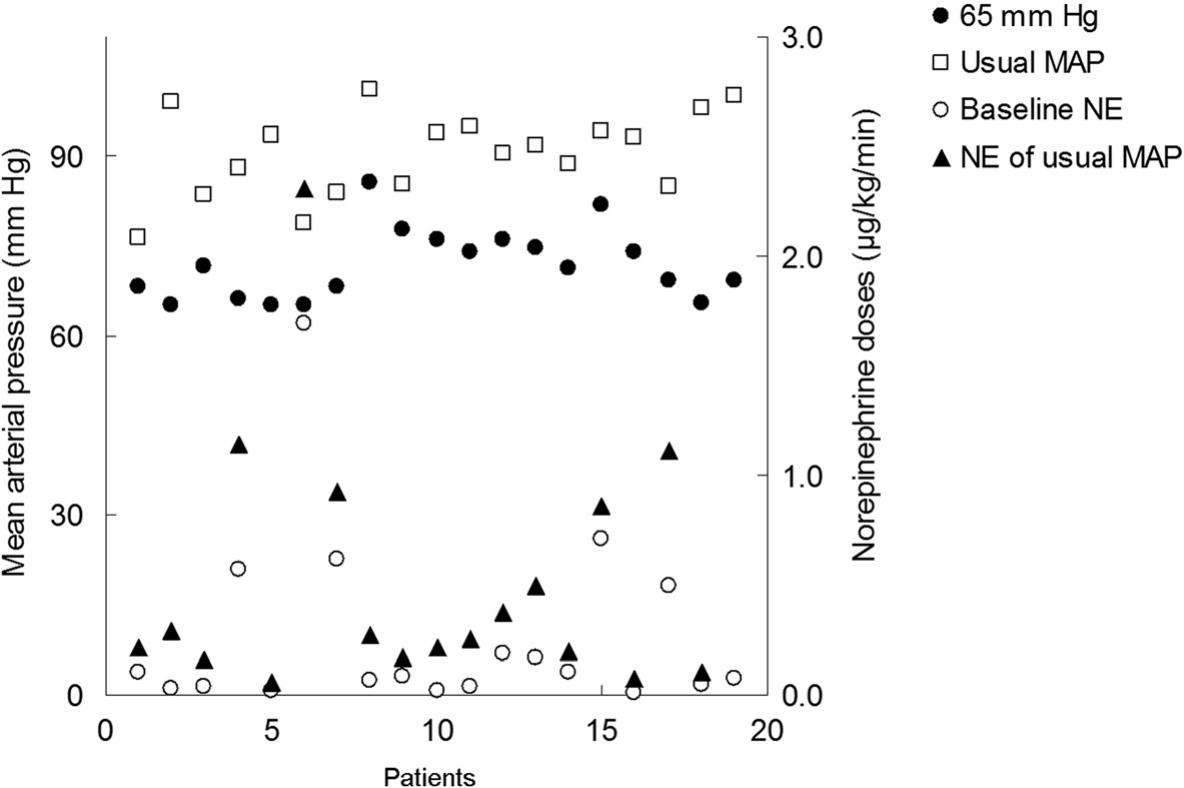
**Baseline and target mean arterial pressure as well as baseline and final norepinephrine doses are displayed.** MAP, mean arterial pressure. NE, norepinephrine.

Increasing MAP from 65 mmHg to target level (from 71 ± 8 to 91 ± 7 mmHg, *P* < 0.001) by augmenting NE dose was associated with significantly increased CVP (from 11 ± 4 to 13 ± 4 mmHg, *P* = 0.002), cardiac output (from 5.4 ± 1.4 to 6.4 ± 2.1 l/minute, *P* = 0.001) and central venous oxygen saturation (from 81 ± 7 to 83 ± 7%, *P* = 0.001) (Table 2, Figure 1).

**Table 2:** **Hemodynamic variables**

	**MAP = 65 mmHg**	**Usual MAP**	***P***
Norepinephrine dose (μg/kg/minute)	0.08 (0.04-0.34)	0.25 (0.16-0.68)*	<0.001
Systolic arterial pressure (mmHg)	107 ± 19	140 ± 15*	<0.001
Diastolic arterial pressure (mmHg)	53 ± 7	66 ± 9*	<0.001
Mean arterial pressure (mmHg)	71 ± 8	91 ± 7*	<0.001
Heart rate (beats/minute)	100 ± 24	99 ± 21	0.793
Central venous pressure (mmHg)	11 ± 4	13 ± 4*	0.002
Cardiac output (l/minute)	5.4 ± 1.4	6.4 ± 2.1*	0.001
Stroke volume (ml)	57.5 ± 21.2	67.0 ± 26.7*	0.003
Urine output (ml/hour)	56 ± 6	55 ± 6	0.921
Hemoglobin (g/dl)	9.4 ± 1.9	9.5 ± 2.0	0.605
Arterial pH	7.38 ± 0.06	7.39 ± 0.06	0.305
Lactate (mmol/l)	2.3 ± 2.3	2.2 ± 2.3	0.598
Arterial partial pressure of oxygen (mmHg)	132 ± 37	142 ± 51	0.250
Arterial partial pressure of carbon dioxide (mmHg)	30 ± 6	29 ± 6	0.303
Arterial hemoglobin saturation (%)	97.4 ± 1.8	98.0 ± 1.6	0.129
PaO_2_:FiO_2_ ratio (mmHg)	259 ± 99	291 ± 104*	0.003
Central venous hemoglobin saturation (%)	81 ± 7	83 ± 7*	0.001

*P < 0.05 versus baseline of mean arterial pressure 65 mmHg. MAP, mean arterial pressure; PaO_2_:FiO_2_, arterial oxygen partial pressure to fractional inspired oxygen. Data presented as means ± SD or median (interquartile range).

### Effect on sublingual microcirculation

Although the vascular density and heterogeneity index were not significantly altered, there were significant increases in small perfused vessel density (from 10.96 ± 2.98 to 11.99 ± 2.55 vessels/mm^2^, *P* = 0.009), proportion of small perfused vessels (from 85 ± 18 to 92 ± 14%, *P* = 0.002), and small MFI (from 2.45 ± 0.61 to 2.80 ± 0.68, *P* = 0.009) compared with a MAP of 65 mmHg (Table 3).

**Table 3:** **Sidestream dark field variables**

	**MAP = 65 mmHg**	**Usual MAP**	***P***
Small vessel density (vessels/mm^2^)	11.29 ± 2.18	11.91 ± 2.14	0.129
Total vessel density (vessels/mm^2^)	12.35 ± 2.29	12.76 ± 2.09	0.227
Small perfused vessel density (vessels/mm^2^)	10.96 ± 2.98	11.99 ± 2.55*	0.009
Total perfused vessel density (vessels/mm^2^)	11.34 ± 3.03	12.24 ± 2.55*	0.021
Proportion of small perfused vessels (%)	85 ± 18	92 ± 14*	0.002
Proportion of total perfused vessels (%)	92 ± 18	96 ± 12*	0.029
Small microvascular flow index	2.45 ± 0.61	2.80 ± 0.68*	0.009
Total microvascular flow index	2.70 ± 0.52	3.05 ± 0.51*	0.002
Heterogeneity index of small diameter vessels	0.59 ± 0.32	0.47 ± 0.27	0.145
Heterogeneity index of total diameter vessels	0.36 ± 0.27	0.30 ± 0.19	0.330

*P < 0.05 versus baseline of mean arterial pressure 65 mmHg. MAP, mean arterial pressure. Data presented as means ± SD.

**Figure 2:**
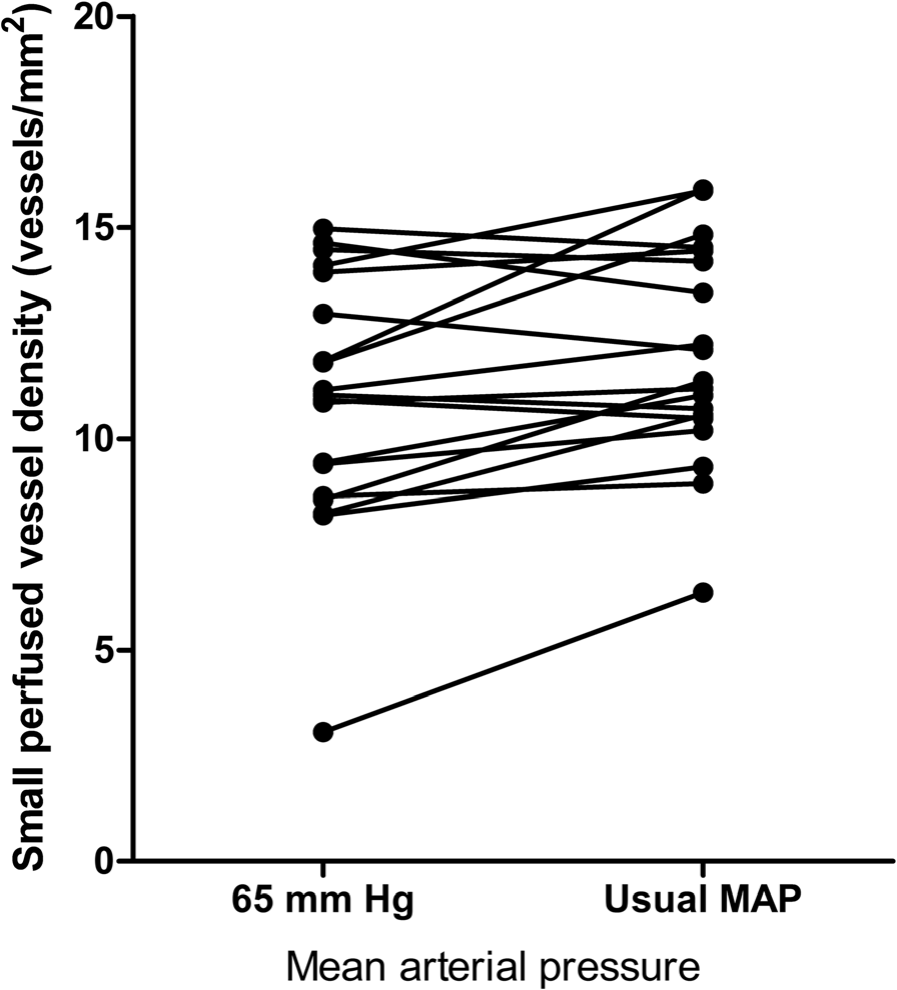
**Results of small perfused vessel density are shown as the mean arterial pressure increased from 65 mmHg to usual level with norepinephrine.** MAP, mean arterial pressure.

**Figure 3:**
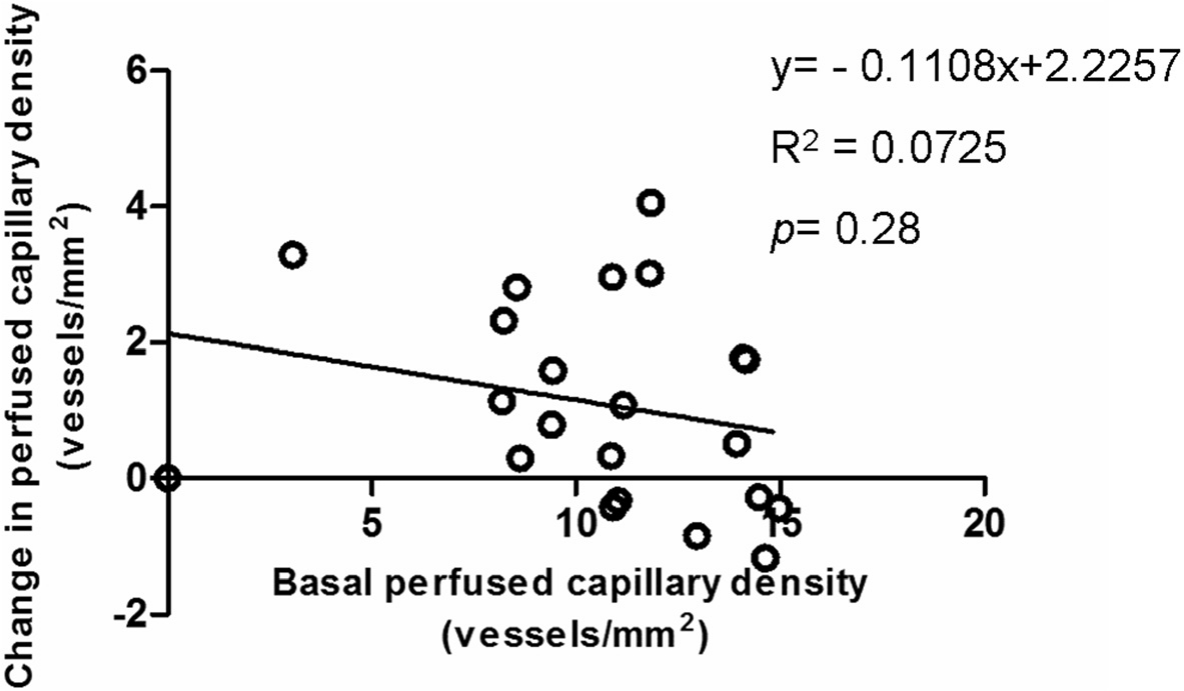
**Relationship between the changes in small perfused vessel density, when mean arterial pressure increased from 65 mmHg to usual levels, with the basal small perfused vessel density at a mean arterial pressure of 65 mmHg.**

**Figure 4:**
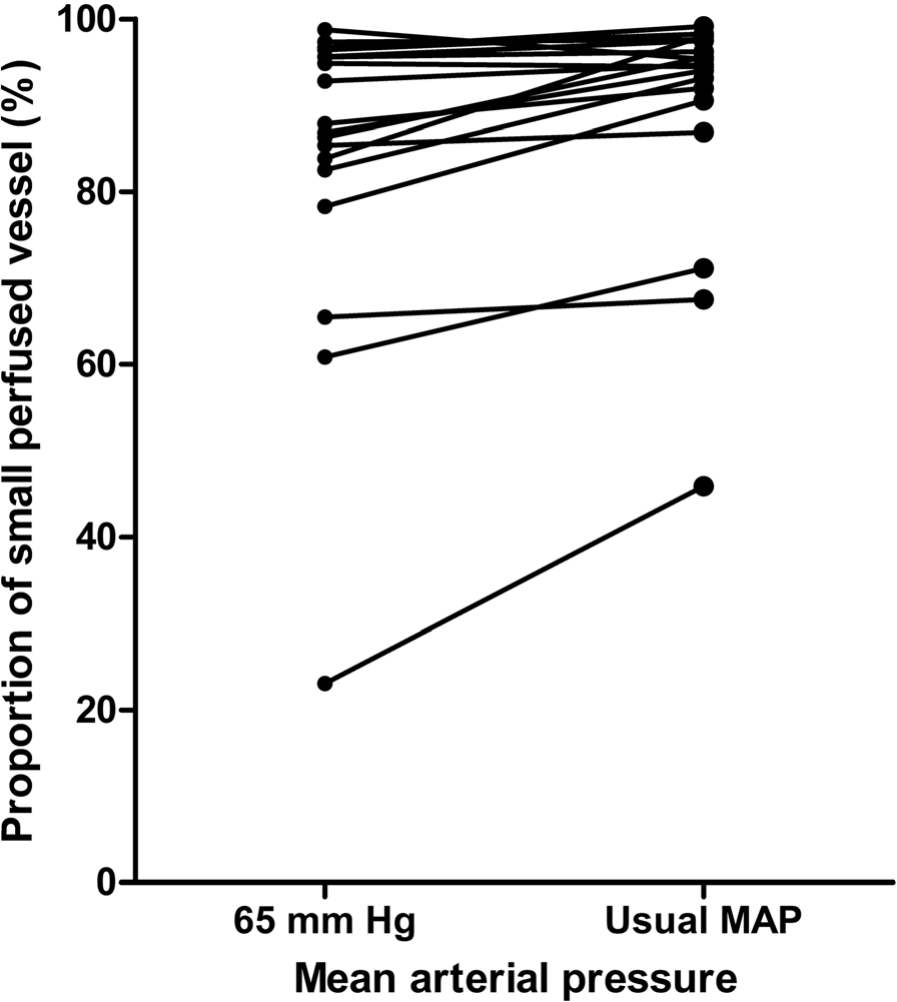
**Results of proportion of small perfused vessels are shown as the mean arterial pressure increased from 65 mmHg to usual levels with norepinephrine.** MAP, mean arterial pressure.

**Figure 5:**
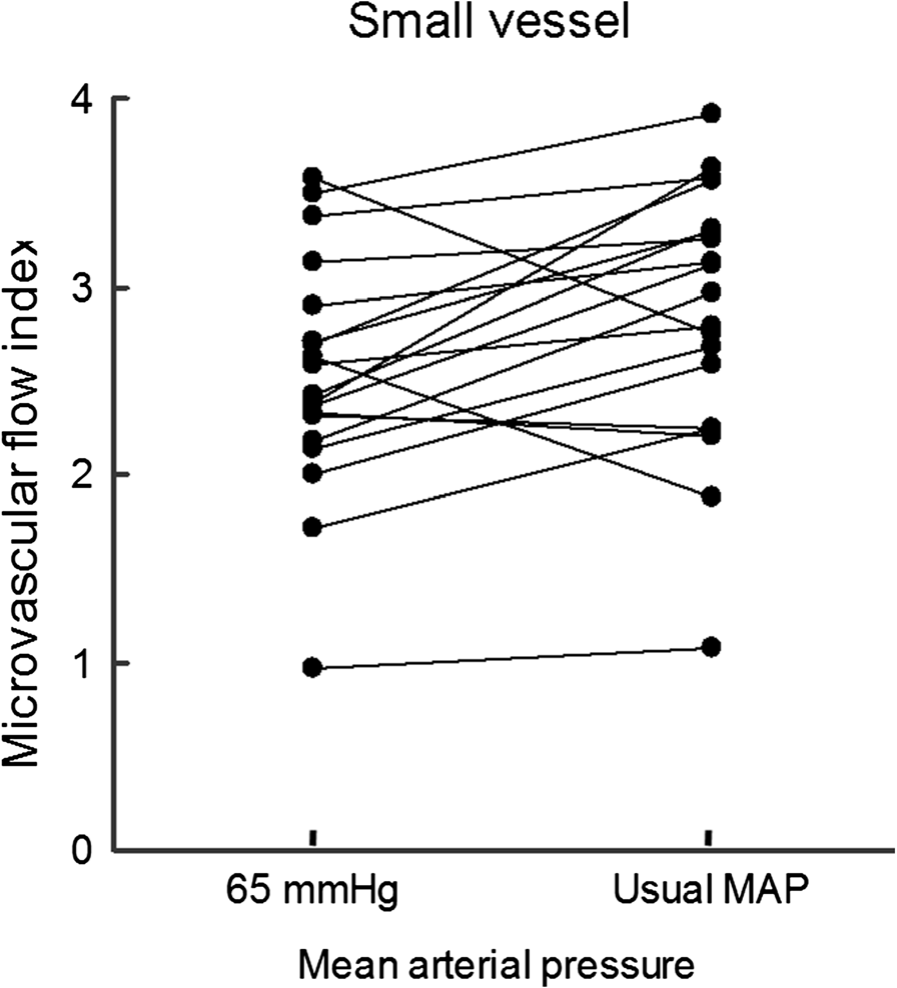
**Results of microvascular flow index are shown as the mean arterial pressure increased from 65 mmHg to usual levels with norepinephrine.** MAP, mean arterial pressure.

Meanwhile, little interindividual variability in small perfused vessel density (Figures 2 and 3), proportion of small perfused vessels (Figure 4) or MFI (Figure 5) appeared (see Additional file 3, a table listing the relationship between the changes of each microvascular variables and basal microvascular parameters).

## Discussion

The main finding of our study suggested that increasing MAP to the usual levels in early resuscitated hypertensive septic shock patients was associated with considerable microcirculatory improvements, characterized by increased perfused vessel density, a higher proportion of small perfused vessels and elevated MFI. Meanwhile, little individual variability developed.

Microcirculatory dysfunction plays an important role in the development of tissue hypoperfusion in patients with septic shock. Although early goal-directed therapy is reported to improve their outcome [2], the routine global hemodynamic parameters such as cardiac output, central or mixed venous oxygen saturation do not work as an effective perfusion index in most cases, and organ failure is still present after rapid correction of global hemodynamic alteration [6]. The microcirculation is responsible for regulating tissue perfusion to meet oxygen requirements and is associated with the development of organ dysfunction. Therefore, it is important to monitor and manage the microcirculation in order to augment perfusion in patients with septic shock.

An adequate MAP is typically essential to restore effective perfusion pressure and organ perfusion in septic shock patients. As the most common pre-existing condition in patients with septic shock [16], the Surviving Sepsis Campaign guidelines [15] have recommended a higher, but vague, blood pressure for patients with previous hypertension. Moreover, MAP targets in clinical trials varies substantially, ranging from 60 to 110 mmHg [19]. Few trials have focused on the effect of different MAP targets in septic shock patients with previous hypertension, let alone which MAP levels should be targeted to improve microcirculation.

Hypertension could cause a rightward shift in cerebral pressure-flow autoregulation [20], which suggests a higher MAP should be targeted in patients with these conditions to maintain organ perfusion. Moreover, a recently published study [16] has observed less renal replacement therapy in the high-target group in septic shock patients with previous hypertension, suggesting that higher MAP might reduce hypoperfusion and organ failure in these patients. However, few studies have looked at the microvascular responses to higher MAP titration in septic shock patients with previous hypertension.

In our study, increasing MAP from 65 mmHg to the usual levels of the patients resulted in improved microcirculatory function in these septic shock patients with previous hypertension, although the cause and effect are unclear. NE was used to titrate MAP to the usual level. As the key vasoactive agent recommended for restoring MAP in the treatment of septic shock, NE might not only perform as the vasopressor but also affect preload and tissue perfusion. Increasing the dose of NE significantly augments cardiac output by 11 to 17% [21], suggesting NE might recruit some blood from the large venous unstressed volume as another method of “endogenous fluid challenge”. Since fluid challenge is demonstrated to improve microvascular perfusion in the early phase of septic shock [22,23], NE-induced microcirculatory improvement might occur because of its role on arterial pressure preservation and blood recruitment.

An NE-induced cardiac output effect might be another reason for the improvement in the microcirculation: rapidly achieving a sufficient perfusion pressure could increase cardiac output in septic shock patients [24]. Georger and colleagues [25] found that NE administration is associated with an increase in MAP and cardiac index, which resulted in improvements of near-infrared spectroscopy variables in septic shock patients. In our study, the NE-induced increase in cardiac output was 1 l/minute on average, which might be another reason for the microcirculatory improvement.

In our study, fluid resuscitation was performed according to the guidelines to maintain a CVP of more than 8 mmHg and central venous oxygen saturation of more than 70% before patients were enrolled, and the basal measurements were taken after a MAP of 65 mmHg was achieved by fluids and NE. This differs from other studies where baseline measurements were taken in the unresuscitated septic shock patients. As a result, the sublingual microcirculation at baseline in our study seems to be less altered when compared with other studies. However, they were similar to microcirculatory parameters in resuscitated septic shock patients [11,12,22].

Considerable microcirculatory interpatient variations are reported in septic shock patients after MAP titration despite fluid resuscitation [12,26], which indicates that the level of MAP should be adapted in consideration of the interindividual effect. In this study, titration to a pressure closer to that of a given patient’s baseline aimed to reduce the interindividual variability, and reveals the microcirculatory alterations after MAP titration to a higher level in septic shock patients with previous hypertension.

### Limitations

Our study has some limitations. Firstly, cardiac output was monitored by the NICOM system to minimize operative invasion. Although the most widely trusted technology for measuring cardiac output is still bolus thermodilution, NICOM is found to have similar monitoring capabilities compared with bolus thermodilution [27,28]. Secondly, the target MAP level was obtained from previous physical examination records or was given by relatives. Although patients with chronic hypertension often undergo more blood pressure measurements in daily life than normotensive patients, the target might be over- or underestimated. Moreover, higher doses of NE may be associated with arrhythmias and other adverse events, which were not observed in our study. Last, but not the least, no assessment of fluid responsiveness was undertaken in our study. NE might have different effects on the microcirculation in patients with or without fluid responsiveness.

It is also important to note that this study is not a controlled study, and lacks a control group and a return to baseline blood pressure. Each patient, therefore, served as his/her own control when increasing MAP from 65 mmHg to the usual level. In addition, the lack of normotensive controls makes the results difficult to extend to normotensive subjects. However, there are several studies [11,12] focusing on the role of different MAP targets in normotensive patients with septic shock. In a large multicenter, open-label trial [16], targeting a MAP of 80 to 85 mmHg, as compared with 65 to 70 mmHg, resulted in no significant differences in mortality for septic shock patients.

## Conclusions

In this open-label intervention study, increasing MAP from 65 mmHg to the usual level in patients was associated with improved microcirculatory function in septic shock patients with previous hypertension.

## Key messages

- Increasing MAP from 65 mmHg to the usual level in patients by NE was associated with increased CVP, cardiac output and central venous oxygen saturation in hypertensive septic shock patients.
- Increasing MAP from 65 mmHg to the usual level in patients by NE was associated with increased small perfused vessel density, proportion of small perfused vessels, and small microvascular flow index in hypertensive septic shock patients.

## Additional files

Additional file 1:
**Study design: predetermined safety limits.**
Additional file 2:
**Microcirculatory measurements and analysis: detailed microcirculatory measurements and analysis.**
Additional file 3:
**Results: relationship between the changes of each microvascular variable and basal microvascular parameters.**

## Abbreviations

CVP: central venous pressure
MAP: mean arterial pressure
MFI: microcirculatory flow index
NE: norepinephrine
SDF: sidestream dark field
