# Supplementary material for: A high mean arterial pressure target is associated with improved microcirculation in septic shock patients with previous hypertension: a prospective open label study
Source: Crit Care. 2015 Mar 30;19(1):130. doi: 10.1186/s13054-015-0866-0 (PMC4409762; doi:10.1186/s13054-015-0866-0)
Supplement: Additional file 1: — Study design: predetermined safety limits. [file 13054_2015_866_MOESM1_ESM.doc]

**A high mean arterial pressure target is associated with improved microcirculation in septic shock patients with previous hypertension: a prospective open label study**

Jing-Yuan Xu, Si-Qing Ma, Chun Pan, Hong-Li He, Shi-Xia Cai, Shu-Ling Hu, Ai-Ran Liu, Ling Liu, Ying-Zi Huang, Feng-Mei Guo,

Yi Yang, Hai-Bo Qiu

**Materials and methods**

Study design

Predetermined safety limits which leading to terminate the research by the treating intensivist were as follows:

1. Life threatening or poorly tolerated arrhythmia.
2. Suspected myocardial infarction which indicated by typical clinical manifestation and electrocardiographic changes.
3. Bleeding, especially the bleeding of the surgical site in postoperative patients.
