# Supplementary material for: A high mean arterial pressure target is associated with improved microcirculation in septic shock patients with previous hypertension: a prospective open label study
Source: Crit Care. 2015 Mar 30;19(1):130. doi: 10.1186/s13054-015-0866-0 (PMC4409762; doi:10.1186/s13054-015-0866-0)
Supplement: Additional file 2: — Microcirculatory measurements and analysis: detailed microcirculatory measurements and analysis. [file 13054_2015_866_MOESM2_ESM.doc]

**A high mean arterial pressure target is associated with improved microcirculation in septic shock patients with previous hypertension: a prospective open label study**

Jing-Yuan Xu, Si-Qing Ma, Chun Pan, Hong-Li He, Shi-Xia Cai, Shu-Ling Hu, Ai-Ran Liu, Ling Liu, Ying-Zi Huang, Feng-Mei Guo,

Yi Yang, Hai-Bo Qiu

**Materials and methods**

Microcirculatory measurements and analysis

Detailed microcirculatory measurements and analysis were as follows:

Measurements of the sublingual microcirculation were obtained using a Sidestream Dark Field with five-fold magnification objective (SDF, Microscan, Microvision Medical, Amsterdam, The Netherlands). The SDF probe was placed under the sublingual area without pressure after removal of secretions. Video sequences of 20 seconds each were recorded from three different sublingual sites^[1]^. These images were stored and later renumbered by an identifier who was blinded to the trial^[2]^. Videos were converted to audio video interleaved (AVI) file format with video processing software (Automated Vascular Analysis 3.0, Academic Medical Center, Amsterdam, The Netherlands), and analysis for each image was performed by two different investigators. Finally, all the variables were averaged to yield a single value for statistical analysis.

Based on the principle that density of the vessels is proportional to the number of vessels crossing arbitrary lines, De Backer score^[3]^ measured variables including vascular density, proportion of perfused vessels and perfused vessel density. Large (mostly venules) and small (mostly capillaries) vessels were separated by a cut-off value of 20 μm in diameter. Three equidistant horizontal and three equidistant vertical lines were drawn on the screen. Defined as the number of vessels crossing these lines divided by the total length of the lines, the vascular density was calculated. The type of flow was defined as no flow, intermittent, sluggish, continuous. Vessel perfusion was categorized visually as continuous (continuous flow for at least 20 seconds), sluggish (decreased but continuous flow for 20 seconds), intermittent (no flow for less than 50% of the time), no flow (no flow for at least greater than or equal to 50% of the time). The proportion of perfused vessels was calculated as follows: 100 × (total number of vessels – no flow –intermittent flow) / total number of vessels. As an important variable with the greatest influence on perfusion^[2]^, perfused vascular density was calculated by multiplying vessel density by the proportion of perfused vessels.

The microcirculatory flow index (MFI) based on determination of the predominant type of flow in four quadrants for small vessels and total vessels were determined with a semi-quantitative methodology. Flow was characterized as no flow = 0, intermittent = 1, sluggish = 2, continuous = 3 to reflect blood velocity. The types of flow were identical to that described in consensus conference recommendations^[2]^. MFI was calculated for all quadrants of the image and averaged for each sublingual site.

Heterogeneity index (HI) was calculated as the difference between highest MFI minus the lowest MFI divided by the mean flow velocity at a single time point^[2, 4]^.

**References**

[1] Morelli A, Donati A, Ertmer C, Rehberg S, Kampmeier T, Orecchioni A, D'Egidio A, Cecchini V, Landoni G, Pietropaoli P, Westphal M, Venditti M, Mebazaa A, Singer M: **Microvascular effects of heart rate control with esmolol in patients with septic shock: a pilot study.** *Crit Care Med* 2013, **41**: 2162-2168.

[2] De Backer D, Hollenberg S, Boerma C, Goedhart P, Buchele G, Ospina-Tascon G, Dobbe I, Ince C: **How to evaluate the microcirculation: report of a round table conference.** *Crit Care* 2007, **11**: R101.

[3] De Backer D, Creteur J, Preiser JC, Dubois MJ, Vincent JL: **Microvascular blood flow is altered in patients with sepsis.** *Am J Respir Crit Care Med* 2002, **166**: 98-104.

[4] Trzeciak S, Dellinger RP, Parrillo JE, Guglielmi M, Bajaj J, Abate NL, Arnold RC, Colilla S, Zanotti S, Hollenberg SM; Microcirculatory Alterations in Resuscitation and Shock Investigators: **Early microcirculatory perfusion derangements in patients with severe sepsis and septic shock: relationship to hemodynamics, oxygen transport, and survival.** *Ann Emerg Med* 2007, **49**: 88-98, 98.e1-2.
