# Supplementary material for: A high mean arterial pressure target is associated with improved microcirculation in septic shock patients with previous hypertension: a prospective open label study
Source: Crit Care. 2015 Mar 30;19(1):130. doi: 10.1186/s13054-015-0866-0 (PMC4409762; doi:10.1186/s13054-015-0866-0)
Supplement: Additional file 3: — Results: relationship between the changes of each microvascular variable and basal microvascular parameters. [file 13054_2015_866_MOESM3_ESM.doc]

**A high mean arterial pressure target is associated with improved microcirculation in septic shock patients with previous hypertension: a prospective open label study**

Jing-Yuan Xu, Si-Qing Ma, Chun Pan, Hong-Li He, Shi-Xia Cai, Shu-Ling Hu, Ai-Ran Liu, Ling Liu, Ying-Zi Huang, Feng-Mei Guo,

Yi Yang, Hai-Bo Qiu

**Results**

Relationship between the change of each microvascular variable and basal microvascular parameter.

|  | Linear relationship  Basal microvascular parameter  R^2^ | *p* |
| --- | --- | --- |
| Change in small vessel density | 0.028 | 0.457 |
| Change in proportion of small perfused vessel | 0.174 | 0.063 |
| Change in small microvascular flow index | 0.017 | 0.561 |
